# Delusional disorder during pregnancy. A case report.

**DOI:** 10.1192/j.eurpsy.2023.2273

**Published:** 2023-07-19

**Authors:** M. Palomo Monge, M. V. Lopez Rodrigo, C. Garcia Montero, M. F. Tascon Guerra, A. Osca Oliver, A. Duque Dominguez

**Affiliations:** 1 Psychiatry, hospital nuestra señora del prado, Talavera de la Reina; 2 Psychiatry, hospital provincial de Ávila, Avila, Spain

## Abstract

**Introduction:**

We present the case of a 34-year-old female patient, 35 weeks pregnant and previously diagnosed with delusional disorder.

**Objectives:**

Somatic personal history: NAMC. intrinsic asthma. Cutaneous psoriasis.

Personal psychiatric history: In psychiatric follow-up since childhood, due to eating problems. Subsequently by adaptive pictures, with anxiety and dysfunctional personality traits intermittently. She resumes contact again in February 2017 presenting frank delusional clinic. Father diagnosed with schizophrenia.

Personal data: 34-year-old woman, married, with a 6-year-old son.

**Methods:**

Current illness: The patient presents active delusional symptoms of about 3 years of evolution, she reports that she knows that there are people in her neighborhood who want to harm her and have guns with which they are constantly shooting to kill her "I hear the shots every day, I have the windows covered with metal plates and I cannot go out with my son, nor to the park, nor to do the shopping”. When she began the delusional symptoms, she was prescribed treatment with olanzapine without response, later with paliperidone palmitate, without response, and then with oral aripiprazole and depot 400mg once a month, with partial response. Prior to the current pregnancy, treatment with clozapine was considered, which the patient accepted but did not tolerate and had to be withdrawn.

**Results:**

Evolution: The patient then remains in treatment with depot aripiprazole, with a partial response and less behavioral repercussion of the delusional content, but with a torpid evolution and tending to chronicity. During this course the patient accidentally becomes pregnant again. The doses of benzodiazepines that she was previously taking to control anxiety and sleep were lowered, maintaining treatment with depot aripiprazole, reducing the dose to 300mg monthly. The pregnancy has proceeded normally to date, with close controls by the gynecology service and monthly visits to psychiatry clinics.

**Conclusions:**

Clinical judgment: Persistent delusional disorder.

In this case, the need arises to maintain depot antipsychotic treatment in a patient with a severe mental disorder during pregnancy, given the serious consequences of delusional content on the patient’s functioning and thus be able to preserve stability at this level during pregnancy.

**Disclosure of Interest:**

None Declared

